# The Tinnitus Retraining Therapy Trial (TRTT): study protocol for a randomized controlled trial

**DOI:** 10.1186/1745-6215-15-396

**Published:** 2014-10-15

**Authors:** Roberta W Scherer, Craig Formby, Susan Gold, Sue Erdman, Charles Rodhe, Michele Carlson, Dave Shade, Melanie Tucker, Lee McCaffrey Sensinger, Gordon Hughes, George S Conley, Naomi Downey, Cynthia Eades, Margaret Jylkka, Ada Haber-Perez, Courtney Harper, Shoshannah Kantor Russell, Benigno Sierra-Irizarry, Mark Sullivan, Tinnitus Retraining Therapy Trial Research Group

**Affiliations:** Johns Hopkins Bloomberg School of Public Health, Baltimore, MD USA; University of Alabama, Tuscaloosa, AL USA; Columbia, MD USA; Jensen Beach, FL USA; National Institute on Deafness and Other Communication Disorders, Bethesda, MD USA; Naval Medical Center, Portsmouth, VA USA; David Grant Medical Center, Travis Air Force Base, Travis, CA USA; Walter Reed National Military Medical Center, Bethesda, MD USA; Wilford Hall Ambulatory Surgical Center, Lackland Air Force Base, San Antonio, TX USA

**Keywords:** Randomized clinical trial, Tinnitus, Tinnitus retraining therapy

## Abstract

**Background:**

Subjective tinnitus is the perception of sound in the absence of a corresponding external sound for which there is no known medical etiology. For a minority of individuals with tinnitus, the condition impacts their ability to lead a normal lifestyle and is severely debilitating. There is no known cure for tinnitus, so current therapy focuses on reducing the effect of tinnitus on the patient’s quality of life. Tinnitus retraining therapy (TRT) uses nonpsychiatric tinnitus-specific educational counseling and sound therapy in a habituation-based protocol to reduce the patient’s tinnitus-evoked negative reaction to, and awareness of, the tinnitus, with the ultimate goal of reducing the tinnitus impact on the patient’s quality of life. Some studies support the efficacy of TRT, but no trial to date has compared TRT with the current standard of care or evaluated the separate contributions of TRT counseling and sound therapy. The Tinnitus Retraining Therapy Trial (TRTT) is a randomized, double-blind, placebo-controlled, multicenter trial for individuals with intolerable tinnitus.

**Methods/design:**

The TRTT is enrolling active-duty and retired military personnel and their dependents with functionally adequate hearing sensitivity and severe tinnitus at US Air Force, Navy, and Army medical centers. Eligible study participants are randomized to TRT, partial TRT, or standard care to determine the efficacy of TRT and its components (TRT counseling and sound therapy). The primary outcome is change in score on the Tinnitus Questionnaire assessed longitudinally between baseline and follow-up (3, 6, 12, and 18 months following treatment). Secondary outcomes include subscale score changes in the Tinnitus Questionnaire, overall and subscale score changes in the Tinnitus Functional Index and Tinnitus Handicap Inventory, and change in the visual analog scale of the TRT Interview Form. Audiological outcomes include tinnitus pitch and loudness match and measures of loudness discomfort levels. The incidence of depression as a safety measure is assessed at each visit using the Beck Depression Inventory Fast Screen.

**Trial registration:**

Clinicaltrials.gov NCT01177137.

## Background

Tinnitus is the perception of sound in the absence of a corresponding external sound for which there is no apparent cause. The precise mechanisms underlying subjective tinnitus are currently unknown. Almost everyone experiences some degree of tinnitus at some time, including sensations of ringing, buzzing, whistling, or hissing. The majority of persons experiencing tinnitus do not report undue distress associated with the tinnitus or require professional help. The prevalence of continuous subjective tinnitus among adults ranges from 10.1 to 14.5% [1], but only about 20% of these individuals require professional help for the treatment of their tinnitus. For some of these individuals, the impact of tinnitus is severely debilitating, impairing their wellbeing and ability to lead a normal lifestyle.

Currently, there is no reliable means of ‘curing’ tinnitus at its source [2, 3]. Evidence from Cochrane reviews of tinnitus treatment trials indicates that no medical or nonmedical treatment is more effective than placebo in eliminating tinnitus. Consequently, current efforts to treat tinnitus focus on reducing the effect of tinnitus on health-related quality of life. Cognitive behavioral therapy, which challenges negative feelings associated with tinnitus and thus seeks to reduce the patient’s focus on tinnitus and the corresponding distress, has some efficacy, as demonstrated in a Cochrane review [4]. Tinnitus retraining therapy (TRT) also aims to reduce the focus of the individual on his or her tinnitus and its impact on health-related quality of life. Tinnitus retraining therapy involves nonpsychiatric tinnitus-specific educational counseling and low-level sound therapy to habituate the patient’s associated negative emotional reactions (for example, annoyance, or anxiety) to tinnitus, the perception of tinnitus (awareness) and, ultimately, the impact of the tinnitus on the patient’s life [5].

Tinnitus retraining therapy is based on a neurophysiological model of tinnitus [6]. The model hypothesizes that the distress associated with tinnitus arises from abnormal subconscious nonauditory mechanisms, mediated primarily by the limbic and autonomic nervous systems. Preliminary evidence from various uncontrolled studies of individuals, who received full or partial TRT as part of their treatment for tinnitus, consistently supports the therapeutic benefits of the full TRT protocol. When the TRT protocol is closely followed, typical rates of clinical success, as judged by a minimum of 20% improvement in two or more impact-on-life scales, approach or exceed 80% improvement [7–12]. A recent Cochrane systematic review of TRT [13] found only one randomized trial that adhered to the TRT protocol as developed by Jastreboff and Hazell [7]. This trial compared TRT with a tinnitus masking intervention [14]. The investigators found that both treatments were efficacious in treating tinnitus over a treatment period of 18 months, but that TRT resulted in greater improvement in patients with greater tinnitus severity at baseline. However, the Cochrane reviewers noted the presence of design flaws that could have resulted in biased findings. Results from three smaller trials have subsequently been published. Bauer and Brozoski [15] found that both TRT and general counseling were efficacious in reducing the annoyance and impact of tinnitus over 18 months, and that the effect size was greater for TRT (1.13) than for general counseling (0.78). In a second trial [16], a comparison of acceptance and commitment therapy with TRT showed that acceptance and commitment therapy yielded a significantly larger reduction in tinnitus impact than did TRT at 10 weeks, 6 months, and 18 months of treatment. Lastly, Tyler *et al.* [17] evaluated masking versus TRT sound therapy and no sound therapy in study participants provided with an equivalent to TRT counseling. This trial reported a positive effect of either sound therapy or masking compared with counseling alone, but had a high proportion of dropouts in all treatment arms [17].

Thus, although the current literature provides some support for the efficacy of TRT, no trial has compared TRT with the current standard of care. The existing trials all compared TRT with a treatment with unknown efficacy. In addition, no trial has evaluated the separate contributions of the component parts of TRT, specifically tinnitus-specific educational counseling and sound therapy. To address these gaps, we are conducting a randomized, placebo-controlled, multicenter trial of TRT, the Tinnitus Retraining Therapy Trial (TRTT), by comparing TRT with the current standard of care in military hospitals, while parsing out the separate effects of TRT counseling and sound therapy. A full description of the rationale for the TRTT has been published previously [18]; this paper describes the design and methods used in the trial.

## Methods/design

The primary objective of the TRTT is to assess the efficacy of TRT as a treatment for severe tinnitus. Our specific goals are to evaluate the efficacy of TRT and its component parts in habituating the perceived magnitude (sensation), perception (awareness), and negative emotional reactions (for example, annoyance, anxiety) to tinnitus, and to measure TRT treatment effect on the impact of tinnitus on each participant’s health-related quality of life.

### Recruitment

The TRTT is currently being conducted at US Air Force, Navy, and Army medical centers with active-duty and retired military personnel and their dependents. Recruitment began in August 2011 at six clinical centers. One center withdrew from recruitment in January 2013, and two additional Army medical centers are currently undergoing certification to participate as clinical centers. As of 1 September 2014, clinical centers have enrolled 136 of the planned 228 study participants.

Eligible study participants have subjective annoying tinnitus of at least one year’s duration for which there is no identified medical etiology. They have normal hearing sensitivity of no worse than mild hearing loss through 4,000 Hz that does not require the use of hearing aids. Eligible participants had not received treatment for their tinnitus within the previous year. Specific inclusion and exclusion criteria include the following:

### Eligibility criteria for TRTT

#### Inclusion criteria

-  Age 18 or older.
-  Primary complaint of continuous, chronic subjective tinnitus for at least one year.
-  Score on the Tinnitus Questionnaire of 40 or more.
-  Functionally normal hearing sensitivity by audiometric thresholds ≤30 dB hearing loss at and below 2000 Hz and ≤40 dB hearing loss at 4000 Hz.
-  Able to understand counseling and comprehend and complete English language questionnaires.
-  Willing and able to give informed consent.

#### Exclusion criteria

-  Predisposing disease with tinnitus symptoms amenable to medical or surgical intervention.
-  Clinical treatment for tinnitus within previous year.
-  Evidence of malingering or exaggeration of tinnitus or hearing symptoms.
-  Emotional, psychological, or psychiatric condition precluding full participation or follow-up.
-  Active involvement in tinnitus-related litigation.
-  Diagnosis of pulsatile tinnitus, somatosounds, or objective tinnitus (that is, spontaneous otoacoustic emissions) that could be responsible for the tinnitus problem.
-  Brain or head trauma requiring treatment 24 months before randomization.
-  Inability to complete audiological testing or other testing required by the clinical trial protocol.

Study candidates must also be willing and able to provide informed consent to allow determination of eligibility and randomization to treatment for the TRTT. Before entry into the TRTT, eligible candidates must confirm willingness to be randomized to an assigned treatment.

### Interventions of the TRTT

In the TRTT, participants have an equal probability of assignment to one of three groups:

1. Full TRT, including tinnitus-specific educational counseling and sound therapy implemented with ear-level sound generators.
2. Partial TRT, including tinnitus-specific educational counseling and sound therapy implemented with placebo sound generators.
3. Standard care, as administered in military hospitals, based on surveys of clinicians at participating clinics and guidelines of the American Speech-Language Hearing Association [19].

Differences between TRT and standard care are itemized in Table 1 and described in detail below.

**Table 1:** **Comparison of tinnitus retraining therapy and standard care in the TRTT**

Topic or concept	Tinnitus retraining therapy (tinnitus counseling and sound therapy)	Standard care
Goal of counseling	Introduce the concept of habituation to facilitate habituation of reaction and secondly, habituation of perception. The goal is for the participant to move to a state where tinnitus is no longer an issue.	Reduce negative cognitive, affective, physical and behavioral reactions to tinnitus; improve the participant’s wellbeing and quality of life.
Short-term goals	Reframe the way the participant views the tinnitus problem by understanding what it is and what it is not; neutralize negative emotional associations by understanding the mechanisms that provoke the emotional reaction to the tinnitus.	Empathize with and validate the participant’s feelings, provide reassurance, promote self-efficacy and engage participant in tinnitus management.
Baseline interview and factors that exacerbate tinnitus	Review completed TRT Interview Form, which informs counselor about impact of tinnitus or decreased sound tolerance on such activities as concentration, sleep, quiet recreational activities, work, restaurant use, sports, concert attendance, or socializing. This information is then discussed during counseling and at follow-up appointments.	Elicit participant’s ‘story’ to identify problem areas.
Description of the anatomy and physiology of the normal and impaired auditory system	Explain structures of outer, middle, and inner ear with special attention to the cochlear structures, function of inner and outer hair cells, and afferent and efferent nerve fibers, using three-dimensional model of an ear, diagrams, and photos. Emphasize that hearing is perceived at the brain, not at the ear, which serves only as a transformer, changing mechanical into electrochemical energy that the brain processes as sound.	Describe in general terms outer and inner ear and some description of nerve pathways.
Processing at the level of the brain	Explain that the cortical area of the brain is responsible for the perception of sound, with monitoring by at least five pre-cortical or subconscious lower levels to filter out irrelevant sounds and enhance new or dangerous signals based on personal characteristics and situation. Describe, with examples, ways in which the brain processes signals, including sensory contrast, selective perception, prioritization, and relationship with tinnitus.	Not described.
Explanation of tinnitus	Explain key points of Jastreboff’s neurophysiological model of tinnitus, using basic charts, to demonstrate the importance of the activation of subcortical, limbic and autonomic systems in tinnitus becoming intrusive.	Provide general description, with assurances that tinnitus is common, does not predict hearing loss, and does not damage health.
Sound therapy	Implement sound therapy with sound generators with low-level, relatively broad-band, sound set to level where sound just begins to mix with tinnitus sound. Recommend environmental sound and to ‘avoid silence’ at all times.	Recommend environmental sound.
Hearing protection	Recommend use of appropriate noise protection in hazardous situations, with caveat that participant should not overuse sound protection. Advise an increase in the volume of the sound generator in slightly noisier settings or the wearing of muffs over sound generators to avoid silence.	Recommend use of appropriate noise protection in hazardous situations. No recommendation for or against routine sound protection.
Sleep	Recommend use of environmental sound throughout the night with tabletop sound machines or sound pillows that have neutral sound that can be adjusted to soft, but audible, volume (not television or talk radio). Discuss etiology of sleep disorders as they relate to tinnitus.	Discuss general guidelines for sleep health and, if necessary for sleep health, recommend use of environmental sound, if an issue. If not an issue, not discussed.
Concentration	Discuss, if an issue.	Discuss ways to shift attention, increase attention span, and avoid distraction, if an issue; if not an issue, not discussed.
Stress	Discuss, if an issue.	Discuss methods to facilitate relaxation, if an issue; if not an issue, not discussed.
Setting goals with individual patient	Discuss making progress towards habituation, making sure the patient understands that it is important not to try to control the process or actively monitor it on a daily basis.	Discuss coping mechanisms that have worked in the past for the participant, including use of environmental sound devices, and strategies to deal with sleep, stress, and concentration. Recommend or decide upon specific strategies for problem areas.
Written materials	Provide written summary of tinnitus counseling and guidelines for sound generator use.	Provide leaflets on tinnitus facts; environmental sound devices; and tips for enhancing sleep, relaxation, and concentration, if these are problem areas; provide list of websites with additional information.

### Tinnitus-specific educational counseling

Tinnitus-specific educational counseling, as implemented in the TRTT, is based on the general principles and methodology described by Jastreboff and Hazell [20], which was the most comprehensive description of the practice of TRT at the time the trial was designed. The overall goals of TRT counseling are to educate the participant about his or her tinnitus condition, initiate tinnitus habituation, and, ultimately, neutralize the participant’s negative emotional associations with his or her tinnitus. The TRT has two primary subgoals: (1) habituation of tinnitus-evoked negative reaction (for example, annoyance, anxiety, or panic related to tinnitus) and (2) habituation of the perception (awareness of tinnitus). Negative emotional reactions arise from distress induced by tinnitus; TRT counseling aims to provide information to reframe (that is, demystify) the participants’ conscious beliefs and begin to neutralize the participant’s negative emotional associations with the tinnitus condition, facilitating habituation of the reaction. Habituation of the perception of the tinnitus signal often occurs after a sufficient level of habituation of the reaction is achieved, and this phase of treatment is facilitated by sound therapy.

The components of the initial TRT counseling session include the following:

-  Explanation of the results of the audiological/tinnitus/hyperacusis clinical evaluation, with an emphasis on the relation of the tinnitus to the clinical findings.
-  Description of the anatomy and physiology of the normal and impaired auditory system, noting that the auditory perception occurs at the level of the brain, not the ear.
-  Discussion of the role of the central auditory system and higher cortical processes to interpret an auditory signal and how this processing relates to the participant’s tinnitus perception.
-  Description of the Jastreboff neurophysiological model of tinnitus [6], which is the centerpiece and focus of TRT counseling in informing the participant about the conscious and subconscious processes involved in the generation and habituation of the tinnitus. These key processes and components of the model as used in the TRTT were summarized by Formby and Scherer [18], and considered in depth by Jastreboff and Hazell [20].
-  Setting goals with and for the individual participant, by discussing progress made towards habituation without trying to control or actively monitor the process.

Participants are counseled individually or with family members. The study audiologist uses a standardized flipchart and visual aids during the TRT counseling session to illustrate and explain the participant’s audiological/tinnitus/hyperacusis results, auditory anatomy and physiology, how the physiological processes relate to tinnitus, the Jastreboff model of tinnitus, and other key concepts of TRT. The counseling audiologist also completes a checklist to ensure practitioner fidelity to the TRTT treatment protocol for tinnitus-specific educational counseling. In the initial TRT counseling session, which lasts for approximately two hours, the audiologist presents information designed to help the participant change the way in which he or she views his or her tinnitus or decreased sound tolerance (hyperacusis), addresses the individual participant’s concerns, and recommends strategies to help the participant achieve the long-term goal of habituation to the tinnitus. Sufficient time is allowed to answer the participant’s questions and to ensure that he or she understands all information presented, including the treatment goal of reducing the impact of tinnitus and, if present, management of decreased sound tolerance, in his or her life.

Follow-up contacts with participants receiving tinnitus-specific educational counseling in the TRTT are held at a second treatment visit one month following the initial counseling session and at 3, 6, 12, and 18 months at the clinical center. Follow-up sessions vary in length from 15 minutes to an hour, depending on the individual participant’s needs; audiologists reinforce topics covered in the initial TRT counseling session and discuss results of follow-up interviews and tests.

### Sound therapy

Sound therapy was also implemented for TRT following the basic principles and methodology outlined by Jastreboff and Hazell [20]. An integral part of TRT, sound therapy aims to facilitate habituation of the perception or awareness of the tinnitus signal by decreasing the contrast between the tinnitus signal and background sounds. Sound therapy is believed to decrease the perception of the tinnitus signal by reducing the gain within the auditory pathways, which further facilitates habituation of the awareness of the tinnitus signal. Sound therapy for TRT may be implemented in any form that achieves an enriched sound environment. The most direct, consistent, and convenient strategy for implementing sound therapy for tinnitus participants with little or no hearing loss is the use of a low-level, relatively broad-band, neutral sound, produced by open-canal bilateral ear-level sound generators. This approach allows sound therapy from the sound generators to be supplemented with environmental sound. We have chosen this method of implementing sound therapy for the TRTT because it allows us to standardize the sound therapy administered and to isolate sound therapy by comparing participants using conventional and placebo sound generators.

In the TRTT, all participants are in TRT Category 1, indicating tinnitus of high severity and no other significant hearing problems; Category 3, indicating the presence of hyperacusis; or Category 4, with hyperacusis as the dominant complaint and exacerbation of symptoms for a prolonged period of time. For the TRTT, hyperacusis was not an exclusion criterion, but a prospective participant with severely debilitating hyperacusis could be excluded at the discretion of the study audiologist if he or she believed that sound therapy was essential for managing the hyperacusis, thus forestalling assignment of the prospective participant to standard care.

The TRTT is using a digital-equivalent sound generator of the Tranquil model, manufactured by General Hearing Instruments, Inc.. Using these sound generators allows standardization of sound therapy across participants and provides the opportunity to achieve a double-blind placebo for sound therapy in the TRTT. General Hearing Instruments, Inc., provide both conventional and placebo sound generators for the TRTT.

The sound generators include data-logging capability to monitor sound therapy use and compliance by the study participant, and sound-level exposure to determine the nature of the primary sound exposure (that is, environmental sound or sound from the sound generators) at any point in time. The sound generator is housed in either a behind-the-ear or an in-the-ear device, both of which offer adjustment of the output volume to a ‘zero’ (fully quiet) noise floor. Placebo sound generators have a physical appearance identical to the conventional instrument so that neither study personnel nor study participants are aware of which sound treatment the participant is receiving. The sound output of the placebo device is designed to be identical to the conventional device upon initial insertion into the outer ear, but after the first 40 minutes of use, the level of sound gradually fades to silence. Double-blinding of the study audiologist and study participant is made possible by a novel re-setting feature of the sound generators. Specifically, the placebo device re-sets within 3 seconds to the full output level, as set at the beginning of initial use, whenever the device is removed from the ear. Thus, if either the study audiologist or study participant re-checks the placebo output sound level, it will be heard again at the same level as was set initially and the decay process will begin anew. This decaying auditory process of the placebo sound generator output mimics normal auditory perceptual adaptation to the ‘sea-shell’ like noise emitted by both the conventional and placebo sound generators. The expectation of this normal perceptual adaptation to the noise is crucial for the success of the placebo sound generator, and reinforced by instructions to all study participants assigned to sound therapy using sound generators.

Fitting and activation of sound generators takes place after the TRT counseling session, and typically requires about one hour. This treatment step includes instruction to the participant on use of and care for the instruments, correct volume settings, schedule of use, and insertion and removal of instruments. The instrument volume is set using programmable software. The audiologist and study participant work together initially to set the sound generator volume for the ear with the most troublesome tinnitus. Then the volume is set for the opposite ear, aiming to match the output of both ears to achieve equal loudness just below the ‘mixing point’. In the TRTT, the ‘mixing point’ is defined as that volume setting at which the noise from the sound generators just starts to mix or blend with the participant’s most bothersome tinnitus sound. The noise from the sound generator should not change the characteristic of the tinnitus, be annoying, or mask the tinnitus. Participants are also instructed not to set the volume of the sound generator too close to the threshold of hearing when first setting the volume, to avoid augmentation of the tinnitus percept, which might occur because of the phenomenon of ‘stochastic resonance’. During the fitting session, the audiologist informs the study participant that the sound generated by the sound generators to achieve effective sound therapy is typically soft, and the noise may not always be heard, especially in noisy environments. Study participants have a range of 6 dB for resetting the output levels of their sound generators, enabling them to adjust the volume for perceived changes in the loudness of the tinnitus from day to day, to maintain a constant level of effective sound. The audiologist routinely instructs the participant to set the volume of the sound generators when they are first inserted into the ears and not to adjust the sound generators afterwards. Study participants are instructed to use the sound generator as much as possible (even all day), or at least for a minimum of 8 hours over the course of the day.

The use of environmental sound enrichment at all times, including when participants use sound generators, is also strongly recommended during TRT, both during tinnitus-specific educational counseling and while fitting the sound generators. Avoidance of silence at all times is a primary tenet of TRT. Exposure to a low level of environmental sound, using a tabletop sound machine, CD player, sound pillow, or other device, preferably with a volume control (so that the volume is not set too low), is emphasized for augmenting sleep when the sound generators are not worn. Use of a television or radio is discouraged because either can attract the attention of the participant and impair initial efforts to fall asleep or stay asleep through the night. Exposure to low-level environmental sound is also recommended during any period of time when the sound generators cannot be worn.

During the second treatment visit and at all subsequent follow-up visits, the audiologist re-checks the sound generator volume settings with the study participant to ensure that the appropriate volume levels are being maintained and the sound generators are functioning properly. In addition, the study participant may come to the clinic at any time between visits, to adjust the sound generator volume setting to maintain the appropriate sound level in relation to his or her tinnitus.

### Standard care

The standard care protocol developed for the TRTT is consistent with information typically provided to participants with severe tinnitus at participating military medical centers and with professional guidelines for tinnitus management, as outlined in the document *Preferred Practice Patterns For the Profession of Audiology*, produced by the American Speech-Language-Hearing Association [19]. Tinnitus management in standard care is based on the individual participant’s complaints, history, audiological evaluation, and self-assessment. Standard care aims to reduce negative cognitive, affective, physical, and behavioral reactions to tinnitus and improve the participant’s wellbeing and health-related quality of life. The following topics are addressed during standard care counseling:

-  Cause, source, and audiological significance of tinnitus.
-  Use of hearing protection in noise.
-  Use of environmental sounds or sound therapy devices (not including ear-level sound generators) to reduce tinnitus perception.
-  Identification of factors that may exacerbate tinnitus.
-  Adaptive coping behaviors.
-  Stress reduction.
-  Strategies to minimize sleep difficulties related to tinnitus.

The audiologist begins standard care by eliciting the participant’s narrative and establishing a mutual understanding of the participant’s tinnitus problem. The audiologist reiterates effective ways in which the participant has coped with tinnitus in the past and emphasizes ways to minimize the tinnitus impact in the future. The discussion is tailored to the individual’s specific complaints, problem situations, coping strategies, personal preferences, and feasibility. Specific topics that may be covered include:

-  *Environmental sounds*, including a demonstration of available devices (for example, CD players with nature sounds, sound pillows), excluding ear-level sound generators or other programmable sound therapy treatments.
-  *Stress reduction*, including a discussion of relaxation exercise programs (for example, Tai Chi, yoga, meditation, progressive muscle relaxation, and visual imagery) with handouts and performance of a short relaxation exercise, such as visual imagery or progressive muscle relaxation.
-  *Sleep*, including a review of normal sleep patterns and factors that affect sleeping, such as stress and emotional upheaval, environmental variables (noise, light, temperature), irregular schedules, jet lag, medications, caffeine, nicotine, and alcohol. The audiologist will assist the study participant in identifying variables that impact his or her specific sleep difficulties and provide a set of recommendations for enhancing sleep.
-  *Concentration* and factors that affect concentration, including noise, temperature, distractions, lighting, hunger, fatigue, health, boredom, stress, depression, and tinnitus. The audiologist will recommend various steps to increase concentration and demonstrate attention-shifting exercises.

Not all standard care topics are covered in detail for all participants; rather, topics are selected based on those issues or problems that affect the individual participant.

Reinforcement counseling is conducted one month after the initial treatment session, to review the specific recommendations that were made during the initial counseling visit and to promote self-efficacy by reinforcing the study participant’s awareness that he or she can manage tinnitus by incorporating those recommendations into daily living. The follow-up counseling session will typically range from 15 to 30 minutes, with the audiologist emphasizing the importance of the consistent use of environmental sound devices and recommended coping strategies for problem situations.

### Monitoring treatment adherence

In the TRTT, audiologists are encouraged to perform both TRT and standard care counseling for a number of reasons. Primarily, we wished to avoid a differential counselor effect that could contribute to the treatment effect in that some audiologists might be better counselors overall than others. Also, we recognized that there could be a possible counselor effect in that some audiologists might prefer to deliver TRT counseling over standard care or vice-versa. Alternatively, an audiologist might be biased *a priori* in the belief that TRT counseling or standard care is the superior treatment. Secondly, we required that at least one audiologist remain blinded to the counseling intervention, that is, the noncounseling audiologist would be blinded and responsible for measuring follow-up audiological or other outcomes. Accordingly, because of scheduling issues and the limited number of audiologists available to participate in the study at some clinics, it was necessary to have all audiologists trained in both counseling methods. The latter was an important consideration for military participation in the TRTT. Lastly, if either type of counseling proved superior, we reasoned that it would be advantageous for the audiologists to have been trained in the superior method so that he or she could offer the counseling in his or her practice subsequent to the completion of the trial.

To maintain the validity of the TRTT, however, it is critical that adherence to protocol is strictly followed for the counseling components of TRT counseling and standard care. Because there is some overlap in the topics covered in the TRT counseling and standard care (for example, recommendation for exposure to ambient low-level environmental noise), there is a real possibility of treatment contamination. Consequently, four procedures were implemented to facilitate compliance with the treatment protocol.

-  Audiologists must be certified to complete either TRT counseling or standard care prior to providing counseling for a study participant in the trial. Certification requires submission of a voice recording that is reviewed and, if acceptable, approved by the protocol monitor. Audiologists providing unacceptable voice recordings cannot conduct counseling sessions in the trial.
-  Audiologists are required to use treatment-specific visual aids during the counseling session that are matched to TRT counseling or standard care.
-  Audiologists must complete a checklist during each study TRT counseling or standard care session by ticking topics covered or discussed in that session.
-  All treatment sessions are voice recorded. Files generated by voice recorders are submitted along with the relevant checklist for review for compliance with the assigned treatment study protocol. Recordings of the first two treatment sessions of each type of counseling conducted by an individual audiologist are reviewed for adherence to protocol. If no deficiencies are noted in these sessions, then one recording from among the next five counseling sessions is randomly selected for review as long as adherence to the treatment protocol is maintained by that audiologist. Nonadherence to the treatment protocol at any one session will be followed by retraining and subsequent review of the next two sessions conducted by that audiologist. Continuing noncompliance results in decertification of that audiologist, who will not be allowed to conduct any further counseling sessions in the trial.

### Randomization

Randomization is stratified by clinical center and occurs in blocks of random multiples of three in the TRTT. The random order of the allocation assignments for each clinic is generated using a computer-generated random permutation. After final determination of eligibility, clinic staff accesses the TRTT website to obtain a randomization assignment, designated either ‘TRT’ or ‘standard care’.

Sound generators are identified in the TRTT using sequential serial numbers, with conventional or placebo sound generators identified by serial number. Prior to the study start, General Hearing Instruments, Inc., provided a block of 600 serial numbers (300 pairs) to the data coordinating center, to identify specific devices to be used in the TRTT. The data coordinating center randomly designated serial numbers to be used for conventional or placebo sound generators, and provided General Hearing Instruments, Inc., with a copy of these assignments. General Hearing Instruments, Inc., then manufactured the standard or placebo sound generators as specified for each assigned serial number.

At the beginning of the study, the TRTT used behind-the-ear devices. General Hearing Instruments, Inc., sent a sufficient number of pairs of sound generators to the data coordinating center to supply clinics with the first six randomized treatment assignments. For assignment to TRT, the data coordinating center placed the sound generators within a box, matching the serial number of the sound generators to the sound generator type (standard or placebo) for randomized treatment assignment (TRT or partial TRT). Standard care assignments comprised a box that contained a sheet of paper noting assignment to standard care. Early in the trial, it became obvious that for some study participants, the ‘in-use’ sensor detection technology for the behind-the-ear devices failed at an unacceptable rate for both conventional and placebo sound generators. The decision was made to move from behind-the-ear sound generators to nonoccluding in-the-ear devices. The data coordinating center continues to send boxed kits to the clinical centers, but instead of including sound generators in the box, the data coordinating center places a form in the box for the clinic to use to order sound generators directly from General Hearing Instruments, Inc.; the form includes the serial number for that assignment. By including the serial number, General Hearing Instruments, Inc., is notified which type of sound generator (conventional or placebo) to manufacture for that study participant, while clinic staff remain unaware of the assignment.

### Outcomes

The TRTT is using patient-reported outcome questionnaires, completed on paper, to assess tinnitus-specific health-related quality of life, general health-related quality of life, and relevant psychological measures. All questionnaires are standardized instruments with well documented psychometric properties. The TRTT also collects information on audiological measures of tinnitus pitch and loudness match and loudness discomfort levels. Additionally, the TRTT assesses the presence of depression at each study visit as a possible adverse event.

The TRTT uses three different tinnitus-specific health-related quality of life measurement tools: the Tinnitus Questionnaire [21]; the Tinnitus Functional Index [22]; and the Tinnitus Handicap Inventory [23]. In addition, the overall impact of tinnitus is assessed using the ten-point visual analog scale of the TRT Interview Form [24]. Each instrument is administered at baseline and at 3-, 6-, 12-, and 18-month follow-up visits. The three tinnitus-specific health-related quality of life instruments (Tinnitus Questionnaire, Tinnitus Functional Index, and Tinnitus Handicap Inventory) are also administered at 30, 42, and 54 months by mail for study participants agreeing to extended follow-up, to assess whether any treatment effect is maintained over time.

The primary outcome measure selected for the TRTT is change in score on the Tinnitus Questionnaire, assessed longitudinally from baseline to the 18-month follow-up visit. The 52-item Tinnitus Questionnaire features a three-point response scale, five subscales, and excellent validity and generalizability properties [21]. The subscales: are psychological distress, intrusiveness, hearing difficulties, sleep disturbances, and somatic complaints. The baseline score of the Tinnitus Questionnaire determines eligibility (score ≥40) for the TRTT and provides a value for comparison at follow-up.

The Tinnitus Functional Index is an instrument recently developed by a group of experienced tinnitus investigators funded by the Tinnitus Research Consortium [22]. It has 25 items and an eight-factor structure, including intrusiveness, reduced sense of control, cognitive interference, sleep disturbance, auditory difficulties (related to tinnitus), relaxation interference, reduced quality of life, and emotional distress. Each question on the instrument has a ten-point response scale (ranging from no effect to extreme effect). The Tinnitus Functional Index has been validated for assessing responsiveness (that is, changes in outcomes related to treatment).

The Tinnitus Handicap Inventory, developed by Newman *et al*. [23], consists of 25 items and has three factors, including functional, emotional, and catastrophic, evaluated using a three-point response scale.

The TRTT will also measure the overall treatment effect of the study interventions using the ten-point visual analog scale of the TRT Interview Form, which asks the study participant to rate the impact of tinnitus on his or her life from ‘not at all’ to ‘as much as you can imagine’.

Because tinnitus may affect the ability of study participants to concentrate, the TRTT is also measuring cognitive function with the Digit Symbol Substitution Test [25]. This test assesses the ability of an individual to focus attention on a task. Indirectly, this measure assesses the ability of the study participant to ignore the tinnitus signal while completing a task requiring attention and psychomotor skill. The Digit Symbol Substitution Test is administered by study staff at baseline, and at the 6- and 18-month visits.

The TRTT collects information using two additional health-related quality of life instruments, the Hearing Handicap Inventory [26] and the EuroQoL [27]. The Hearing Handicap Inventory is a ten-item instrument [26] used in the TRTT to assess the impact of hearing loss on overall and tinnitus-related health-related quality of life. The EuroQoL consists of five items that assess difficulty in performing activities of daily living [27]. It uses a three-part Likert scale and a visual analog scale that ranges from 0 (worst imaginable health) to 100 (best imaginable health state). The Hearing Handicap Inventory and the EuroQoL are completed at baseline and at 6- and 18-months follow-up visits.

The TRTT is administering three psychological instruments, the State-Trait Anxiety Inventory [28], the Positive and Negative Affect Schedule [29], and the Life Events Checklist [30], to assess personality traits or life events that might be associated with treatment efficacy. These instruments are administered at baseline and at 6- and 18-month follow-up visits. In addition, the TRTT is administering the Beck Depression Inventory Fast Screen [31] at baseline and all follow-up visits, as a safety screen for depression.

The State-Trait Anxiety Inventory is a self-report measure of trait anxiety (or anxiety ‘proneness’) and state anxiety (that is, transitory anxiety experienced under specific conditions). Participants complete both subscales of the State-Trait Anxiety Inventory at baseline and the state subscale at 6- and 18-month follow-up visits. The Positive and Negative Affect Schedule consists of 20 adjectives describing feelings or emotions (for example: excited, scared, irritable, nervous), that might change with treatment benefits. To assess life events that could result in stress and anxiety impacting wellbeing and possibly treatment efficacy, the TRTT also administers the Life Events Checklist.

The Beck Depression Inventory Fast Screen is used as a screen for depression. The TRTT requires a threshold score of 4 or more, or endorsement (a response of 2 or 3) of suicidal thoughts or wishes, to determine the need for further mental health evaluation. Repeated measures of the Beck Depression Inventory Fast Screen over the course of the TRTT will permit study participants to be monitored for the onset of serious depression as a possible consequence of treatment or other contributing factors.

### Audiological outcomes

Audiological outcomes measured in the TRTT include pure-tone audiometric thresholds, speech recognition thresholds, tinnitus pitch and loudness match, and loudness discomfort levels. Pure-tone and speech audiometry are used in the TRTT to evaluate changes in the participant’s hearing sensitivity and are performed at baseline and at 6-, 12-, and 18-month follow-up visits. Tinnitus pitch match, which identifies the frequency or frequency range that most closely matches the pitch of the participant’s tinnitus, and tinnitus loudness match, which matches the intensity of the participant’s most troublesome tinnitus, will be performed at baseline, and at 6-, 12-, and 18-month follow-up visits. Loudness discomfort level is a measure of the sound level at which an individual first judges the loudness of a sound to cause discomfort. Loudness discomfort levels set the functional upper limit of the listener’s dynamic range, that is, the range between the hearing threshold and the loudest comfortably tolerated sound. In the TRTT, loudness discomfort levels are used to determine whether intolerance to sound (hyperacusis) is abnormal and associated with the participant’s tinnitus. If hyperacusis is present, the loudness discomfort levels are used to determine the steps required to modify the level of sound set in the sound generators by the audiologist. Loudness discomfort levels are measured at every study visit. The audiologist performing the audiometric tests for an individual study participant is blinded to that participant’s treatment assignment.

### Efficacy outcomes

The primary objective of the TRTT is to assess the efficacy of TRT, achieved through TRT counseling and sound therapy (provided by conventional sound generators), as a treatment for severe debilitating tinnitus. The primary outcome to be measured in the TRTT is the difference in score on the Tinnitus Questionnaire between baseline and follow-up, assessed longitudinally, after 3, 6, 12, and 18 months of treatment. The TRTT also will investigate the efficacy of sound therapy by comparing Tinnitus Questionnaire scores of participants assigned to conventional sound generators with those assigned to placebo sound generators, where both groups are assigned to TRT counseling. Further, the TRTT will investigate the efficacy of TRT counseling by comparing Tinnitus Questionnaire scores in the group of participants assigned to TRT counseling and sound therapy achieved with placebo devices versus Tinnitus Questionnaire scores for those in the group assigned to standard care, with the assumption that the effect of placebo sound therapy is negligible. Secondly, the TRTT will assess the efficacy of treatment by measuring the difference between baseline and end-of-treatment Tinnitus Questionnaire scores in the three treatment groups.

The secondary efficacy outcomes will compare groups by assessing end-of-treatment and longitudinal changes in scores on the subscales of the Tinnitus Questionnaire; total and subscales of the Tinnitus Functional Index and Tinnitus Handicap Inventory; TRT visual analog scale; Digit Symbol Substitution Test; and psychoacoustic measures of tinnitus pitch and loudness match and loudness discomfort levels. Maintenance of treatment efficacy will be assessed by comparing scores on the Tinnitus Questionnaire, Tinnitus Functional Index, and Tinnitus Handicap Inventory between baseline, the 18-month follow-up visit, and each extended follow-up visit at 30, 42, and 54 months.

### Ethics

The TRTT will be conducted following the ethical principles of the Declaration of Helsinki. This study received ethical approval from the institutional review boards of the University of Alabama for the chair’s office and at the Johns Hopkins Bloomberg School of Public Health for the data coordinating center. Institutional review boards approving the protocol and informed consent statement at clinical centers include: the Walter Reed National Military Medical Center institutional review board, the 59th Medical Wing institutional review board (Wilford Hall Ambulatory Surgical Center and David Grant Medical Center), clinical investigation departments at Naval Medical Center San Diego and Naval Hospital Camp Pendleton, and the Naval Medical Center Portsmouth institutional review board. No study participant will be enrolled in the study before informed consent is obtained and documented by a signed informed consent statement.

### Sample size

We based our sample-size calculation on the difference in Tinnitus Questionnaire scores between baseline and 18 months of follow-up (end of treatment). We consulted experts familiar with the Tinnitus Questionnaire to estimate the minimal clinically important difference between study groups. Richard S. Hallam (who developed the Tinnitus Questionnaire) proposed, ‘An effect size of 0.8 or perhaps 1.0 on the emotional distress subscale is a reasonable criterion for [clinically] significant change’, (personal communication). An effect size of 0.8 to 1.0 on the full Tinnitus Questionnaire would correspond to a change of approximately 10 to 13 points. Wolfgang Hiller, a researcher with extensive experience with the Tinnitus Questionnaire stated, ‘A change of less than 5 to 6 points does not mean much’, (personal communication). Based on these considerations, we chose to design the TRTT with sufficient power to detect a ten-point difference for the primary comparison (that is, between TRT and standard care) and a seven-point difference for the secondary comparisons (that is, the separate efficacies of sound therapy and TRT counseling). We assumed a type-I error rate of 0.05, using a two-sided test and 80% power for the secondary comparisons. We further assumed that a standard deviation of 12.5 would hold for all groups and that there would be 10% attrition. Accordingly, by dividing an α of 0.05 among the three comparisons and accounting for 10% attrition, we estimated that a total of 228 study participants (76 participants in each of the three groups) will be required to yield at least 80% power to detect a seven-point difference for analyses of the TRT components, sound therapy and TRT counseling [32]. This sample size provides for greater than 95% power for the primary analysis (TRT versus standard care). Our sample-size estimate is conservative because the primary outcome is a longitudinal analysis, using data from follow-up visits at 3, 6, 12 and 18 months, rather than a difference in score between two time points (that is, baseline and end of treatment at 18 months).

### Data management

The TRTT clinical center staff collect study data on paper data collection forms and enter data online using the TRTT website. All data items are validated against criteria specified in the data dictionary, including checks for consistency with other responses and completeness during data entry. Data errors or inconsistencies are flagged and displayed immediately online during the data entry process. Data flags require ‘immediate correction’ or may be set as ‘warnings’ (missing data). Flags that require ‘immediate correction’ do not permit submission of the form without correction. Warnings will not disallow submission of the form, but must be addressed in a timely manner. Warnings are entered into an error log, and an error report is produced by the system. Any variables that are marked as possibly in error are reported to the clinical centers. In addition, errors are detected by data coordinating center staff during routine audits, in which data on copies of paper data collection forms are compared with data already entered into the database. Sites make corrections in the TRTT website database or verify that the information entered is correct. Any new errors generated during editing are added to the error log. Immediately following completion of entry of all expected data collection forms for an individual study participant, a set of cross-form and cross-visit data queries will be run and the clinic queried for inconsistencies detected. Official interim datasets, containing data entered ‘as of’ a given date, are created for monitoring purposes in conjunction with data and safety monitoring board interim report generation. These datasets are archived for reference in duplicate analysis.

Security measures include password protection for each user who is granted privileges; access is based on ‘need to know’. All elements of the database are backed up daily and weekly onto high-capacity cassette tapes, and monthly backups are transferred to CD media. In addition to data kept on site, copies of weekly backups are stored off site in a fireproof safe.

### Statistical methods

Initial analyses will be descriptive in nature, using means, standard deviations, and proportions to describe baseline characteristics of the study participants, both combined and by intervention group. Distributions of continuous variables will be examined for symmetry, and transformations will be considered for seriously skewed variables. For continuous variables, one-way analysis of variance will be used to compare intervention groups on demographic and other baseline characteristics. For dichotomous or other categorical variables, chi-square tests will be used to compare intervention groups, and enrolled and excluded groups. Pearson correlation coefficients will be calculated to assess the strength of the associations among the various outcome measures and to examine associations of other covariates with outcome measures; potential confounding variables will be considered for inclusion in secondary analyses involving regression models.

The primary outcome variable will be change in the Tinnitus Questionnaire score, using spaghetti plots for initial exploratory analyses and longitudinal data analyses for the primary analytic method. Indicator variables will identify treatment group, with coefficients in the models representing the primary focus on group differences. Mean Tinnitus Questionnaire scores will be compared between two treatment groups using generalized estimating equations. The basic model will account for effects of clinical center, treating audiologist, treatment group, and, possibly, personality traits, as measured by the State-Trait Anxiety Index or Positive and Negative Affect Schedule. Other factors that might be included are scores from the Hearing Handicap Inventory or early completion of treatment. Similar models will be run for the secondary objectives. These variables in the models will be used to estimate the relation between various personality characteristics and successful habituation to tinnitus. Interaction terms between these participant characteristics and treatment groups will be included, to assess whether the effect of TRT is greater for certain types of participant. Similar analyses will assess whether age, sex, or other participant characteristics are predictive of successful habituation with or without TRT.

For the secondary efficacy analyses, we will compare Tinnitus Questionnaire mean scores at 18-months follow-up (end of treatment) among treatment groups using analysis of covariance, adjusting for baseline Tinnitus Questionnaire scores and heterogeneity of tinnitus status in the study population. We will also adjust for important baseline covariates and variables that may be unbalanced at baseline and potentially related to Tinnitus Questionnaire score. We will also use generalized estimating equations for this analysis.

We will use this approach with other outcome variables, including the Tinnitus Questionnaire subscales, global and subscale scores of the Tinnitus Functional Index, Tinnitus Handicap Inventory, and visual analog scale of the TRT Interview Form. We will also compare baseline audiometric measures, psychoacoustic variables of tinnitus pitch and loudness match, and loudness discomfort levels to evaluate change across time in these measures.

All analyses will be based on the principle of intention to treat (that is, in the analysis study participants will be included in the group to which they were randomized) regardless of whether they adhered to, or completed, the assigned treatment. For the primary outcome, the only participants who will not be included will be those for whom Tinnitus Questionnaire could not be assessed at any follow-up visit due to loss to follow-up. However, we anticipate that few, if any, participants will be unable to complete at least one follow-up Tinnitus Questionnaire measure. To account for missing values, we will use multiple imputation, which assumes that missing data are missing at random.

### Organization

Organizational units of the TRTT include a study chair’s office, a data coordinating center, clinical centers, and various committees. The National Institute on Deafness and Other Communication Disorders (NIDCD) at the National Institutes of Health funds the TRTT through a cooperative grant arrangement and provides a project officer. Leadership in the TRTT is shared by the study chair and the director of the data coordinating center.

The chair’s office is responsible for the overall scientific and administrative aspects of the trial. Study personnel at the chair’s office include the study chair, TRT and standard care training and protocol monitors, a human research protocol monitor, and an administrative assistant. The chair’s office is responsible for supervision of the overall study by integrating the activities of the data coordinating center and clinical centers, including recruitment and oversight of the clinical centers. The chair’s office provides assistance to clinical centers on protocol implementation and monitors clinical center institutional review board approval. It is charged with resolution of all technical and scientific questions regarding clinical training and treatment issues and complications. The study chair organizes and conducts TRT and standard care training sessions. Together with the data coordinating center, the study chair implements quality assurance measures to monitor treatment adherence in TRTT clinical centers. The data coordinating center oversees scientific aspects of study design, and serves as the focus of study communications, training, and certification of clinical staff in trial procedures, protocol interpretation, data processing, data analysis, and quality assurance activities. Staff at the data coordinating center include the director, biostatistician, director of information management, database programmer, statistical programmer or analyst, project coordinator, and research assistants. The data coordinating center prepares, maintains, and distributes study documents, data collection forms, and a web-based data system to process data with computer-based randomization schedules for each clinical center. The data coordinating center trains clinical center staff on the study protocol, supervises TRTT data collection and processing, and performs all study analyses. Quality control and assurance responsibilities include certification of clinical center staff and routine monitoring of recruitment, data processing, and adherence to study protocol. The data coordinating center is the official TRTT archive for all study data, and will prepare a public use data set of TRTT data.

Clinical center staff include at least two audiologists, a coordinator, an otolaryngologist, or study physician, and a data system operator. Clinical centers are responsible for participant recruitment, treatment administration, and follow-up according to study protocol. Data management activities at participating clinics include proper completion of all data collection forms, transmission of that information to the data coordinating center using the TRTT website accurately and timely, and responses to requests for data clarification or correction.

The TRTT has three primary standing committees: a steering committee, an executive committee, and a data and safety monitoring board.

Members of the steering committee include the study chair, data coordinating center director, biostatistician, clinical center directors, and NIDCD project officer. The steering committee is responsible for the review and approval of trial procedures, resolution of technical or operational issues, review of study progress and approval of major changes to the study protocol. The steering committee is also responsible for acting on advice from the data and safety monitoring board, appointment or termination of subcommittees, review and approval of ancillary studies, and oversight of publication of study findings.

The executive committee includes the study chair, data coordinating center director, and NIDCD project officer, and is responsible for implementation of decisions made by the steering committee and matters affecting the day-to-day operations of the trial.

The data and safety monitoring board is responsible for ensuring participant safety and monitoring the overall performance of the trial. This board reviews the study data, as they are collected, for evidence of harmful or beneficial treatment effects. The members of this committee, appointed by the NIDCD, include a chair and three additional voting members not affiliated with the study. This committee is the only committee provided with evidence of treatment effects while the study is still in progress. The data and safety monitoring board usually meets twice a year, but additional meetings are scheduled as necessary.

### Dissemination policy

All TRTT papers that describe the main results of the TRTT (that is, comparisons of treatment groups) will follow corporate or group authorship format, naming ‘The Tinnitus Retraining Therapy Trial Research Group’ as author, with individual investigators and clinical center staff acknowledged. No comparison data will be available prior to completion of the study and no clinic may publish data obtained from their clinic independently. Writing committees for TRTT papers will include at least one representative from the chair’s office, one from the data coordinating center, and other study group members based on interest and expertise. All TRTT manuscripts will be submitted to journals complying with the National Institutes of Health Public Access Policy, and all publications will be archived in PubMed Central, as required by this policy. Presentations at conferences describing or presenting TRTT results follow the same guidelines and require clearance by the steering committee.

### Public access to protocol and data

The TRTT data will become available to outside investigators at the conclusion of the trial and following publication of the main study findings. To protect the confidentiality of participant data, only de-identified data will be made available for secondary analysis. All secondary users of data will be asked to sign a data use agreement that stipulates: confidentiality and data security standards must be adhered to by the recipient; data are to be used for research purposes approved by the institutional review board only; no effort will be made to identify individual participants; and data will not be transferred to other users by the recipient.

## Discussion

This report describes a randomized clinical trial that will determine the efficacy of TRT and its component parts, tinnitus-specific educational counseling and sound therapy, compared with standard care, in a military population. Two features of the study highlight efficiency in design. First, we designed the trial with three arms; two arms differ only by whether placebo or conventional sound generators are used and the third arm is standard care. This design allows us to make multiple comparisons and parse out the separate effects of the TRT components. In addition, we are conducting the study in US military hospitals. We chose to conduct the study in this setting given the higher prevalence of tinnitus among individuals exposed to intense noises. Because the military population is similar to the general population in all other aspects, we believe that the study results will be generally applicable.

Because the TRTT is assessing tinnitus-specific health-related quality of life using three commonly used instruments (Tinnitus Questionnaire, Tinnitus Functional Index, and Tinnitus Handicap Inventory), we will be able to assess comparability across these instruments. We will also assess whether the Digit Symbol Substitution Test is a useful measure to assess the ability to concentrate in individuals with tinnitus.

A unique feature of the TRTT is the development of a standardized protocol for both TRT and standard care, as practiced in the military for persons suffering from severe tinnitus. The TRTT developed a standardized tinnitus-specific educational counseling component of TRT, comprised of scripts, a flipchart, and visual aids to be used by all audiologists at all clinical centers. This standardized treatment protocol allowed different audiologists across military centers to learn and administer TRT as a treatment for severe tinnitus not only within the trial, but in their clinical practices. In addition, the TRTT investigators engaged the assistance of General Hearing Instruments, Inc., in the development of a placebo sound generator, enabling measurement of the separate effects of the component parts of the TRT intervention, tinnitus-specific educational counseling and sound therapy. Both placebo and conventional sound generators can be used to assess participant compliance by recording times when the device is worn or not worn. This ability provides for the monitoring of treatment fidelity. The TRTT investigators also developed a standard care protocol to achieve uniformity in the care of tinnitus by surveying clinical centers before the trial began and incorporating current guideline recommendations [19]. Standardization of TRT and standard care for use in a multicenter randomized clinical trial required the essential elements of each intervention to be identified and standardized across different study audiologists and treatment sites. The subsequent streamlining and distillation of the essential components meant that all details were mandated by protocol, but allowed for individual variation by audiologists within strict adherence to the essential components. We hope that standardization of the interventions for the TRTT will facilitate and expedite treatment outcomes in the trial and eventually within the clinical community if either or both TRT and standard care prove efficacious.

## Trial status

The TRTT is currently in the process of recruiting study participants.

## Abbreviations

NIDCD: National Institute on Deafness and Other Communication Disorders
TRT: tinnitus retraining therapy
TRTT: Tinnitus Retraining Therapy Trial.
